# Supplementary material for: Peptidomics as a Tool to Assess the Cleavage of Wine Haze Proteins by Peptidases from Drosophila suzukii Larvae
Source: Biomolecules. 2023 Feb 28;13(3):451. doi: 10.3390/biom13030451 (PMC10046487; doi:10.3390/biom13030451)
Supplement: Supplementary file 1 [file biomolecules-13-00451-s001.zip › supplementary_information.pdf]

# Supplementary Information

## Peptidomics as a tool to assess the cleavage of wine haze proteins by peptidases from *Drosophila suzukii* larvae


## Authors

Wendell Albuquerque[1], Parviz Ghezellou[2], Kwang-Zin Lee[3], Quintus Schneider[1], Phillip Gross[1], Tobias Kessel[3], Bodunrin Omokungbe[3], Bernhard Spengler[2], Andreas Vilcinskas[3,4], Holger Zorn[1,3], Martin Gand[1*]

## Affiliations

[1]Institute of Food Chemistry and Food Biotechnology, Justus Liebig University Giessen, Heinrich-Buff-Ring 17, 35392 Giessen, Germany

[2]Institute of Inorganic and Analytical Chemistry, Justus Liebig University Giessen, Heinrich-Buff-Ring 17, 35392 Giessen, Germany

[3]Fraunhofer Institute for Molecular Biology and Applied Ecology, Ohlebergsweg 12, 35392 Giessen, Germany

[4]Institute for Insect Biotechnology, Justus Liebig University Giessen, Heinrich-Buff-Ring 26-32, 35392 Giessen, Germany

*Corresponding author, E-Mail: Martin.Gand@lcb.chemie.uni-giessen.de, ORC-ID: 0000-0001-8211-691X

**Supplementary Table S1:** Peptide sequences for rTLP and rCHI identified by top-down peptidomics following incubation with peptidases from *Drosophila suzukii* larvae at pH 3.5.

| Protein | Peptide |
|---|---|
| **rTLP** | NVNAGTTGGRVW |
| | |
| **rCHI** | YSGFGNDGSTDANKREIAA |
| | SGFGNDGSTDANKREIAAF |
| | NSYSGFGNDGSTDANKREIAAF |
| | SGFGNDGSTDANKREIAA |
| | NSYSGFGNDGSTDANKREIAA |
| | YSGFGNDGSTDANKREIAAF |
| | SALNSYSGFGNDGSTDANKREIAA |
| | LSALNSYSGFGNDGSTDANKREIAA |
| | WMNNVHSVIGQ |
| | DVVTSFKTAL |
| | NVHSVIGQGFGATIR |
| | LSALNSYSGFGNDGSTDANKREIAAF |
| | DVVTSFKTALW |
| | GFGATIRAING |
| | WMNNVHSVIGQGFGA |
| | YTRAAFL |
| | RVQYYKDY |
| | WMNNVHSVIGQGF |
| | GATIRAING |
| | TDVVTSFKTAL |
| | NKREIAAF |
| | NYGAAGNSIGFNGLSNPGIVAT |
| | FAHVTHETGHF |
| | IEEINGASHN |
| | TIRAINGA |
| | NYGAAGNSIGFNG |
| | WMNNVHSVIGQGFGATIR |
| | IEEINGASHNY |
| | SALNSYSGFGNDGSTDANKREIAAF |
| | FYTRAAF |
| | FFDGIINQ |
| | SVIGQGFGATIRAINGA |
| | NVHSVIGQGF |
| | NYGAAGNSIGFNGLSNPGIVA |
| | NVHSVIGQ |
| | GFGATIRAINGA |
| | FDGIINQ |

| | |
|---|---|
| | VVTSFKTAL |
| | NVHSVIGQGFGA |
| | DVVTSFKT |
| | VVTSFKTALW |
| | FKTALWF |
| | WFWMNNVH |
| | NVHSVIGQGFGATIRAING |
| | |

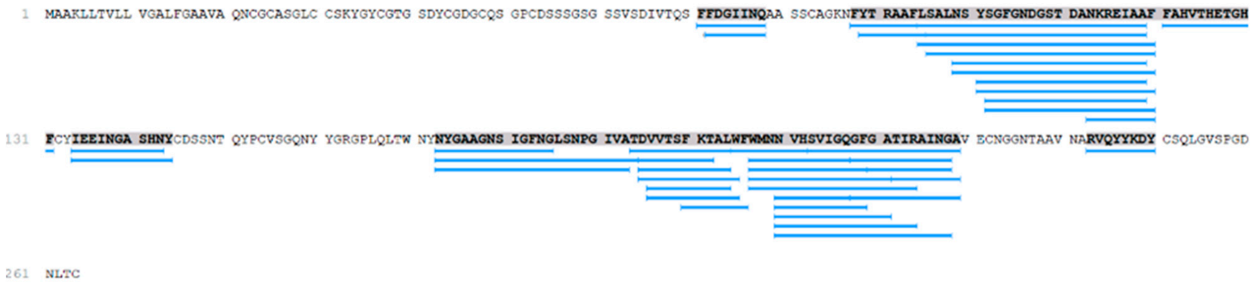


**Supplementary Figure S1:** Peptide products from the cleavage of rCHI (UniProt ID: QX7AU6) following incubation with peptidases from *D. suzukii* larvae. The highlighted amino acid sequences (blue underlined) were identified by *de novo* sequencing and database comparison using Peaks Studio v.X+.

```
  1  ATFNIQNHCS YTVWAAAVPG GGMQLGSGQS WSLNVNAGTT GGRVWARTNC NFDASGNGKC ETGDCGGLLQ CTAYGTPPNT LAEFALNQFS NLDFFDISLV
101  DGFNVPMAFN PTSNGCTRGI SCTADIVGEC PAALKTTGGC NNPCTVFKTD EYCCNSGSCS ATDYSRFFKT RCPDAYSYPK DDQTSTFTCT AGTNYEVVFC
201  P
```

**Supplementary Figure S2:** Peptide products from the cleavage of rTLP (PDB ID: 4JRU) following incubation with peptidases from *D. suzukii* larvae. The highlighted amino acid sequences (blue underlined) were identified by *de novo* sequencing and database comparison using Peaks Studio v. X+.
